# Reactivity of Z-3-Hexenal with Amino Groups Provides a Potential Mechanism for Its Direct Effects on Insect Herbivores

**DOI:** 10.3390/insects16060582

**Published:** 2025-05-31

**Authors:** Jurgen Engelberth, Marie Engelberth

**Affiliations:** Department of Biology, Health, and the Environment, The University of Texas at San Antonio, San Antonio, TX 78249, USA; marie.engelberth@utsa.edu

**Keywords:** Z-3-hexenal, green leaf volatiles, plant defense, insect herbivore, artificial diet, Schiff base formation, aldehyde, binding assay

## Abstract

Green leaf volatiles (GLV) have been shown in the past to significantly affect herbivore performance in plants by activating direct and indirect defense measures. At the same time, insect herbivores have developed multiple mechanisms to reduce the quantities of Z-3-hexenal, the first product of the pathway. Among the various measures is the abundance of a molecule named hexenal trapping molecule (HALT), which appears to bind large quantities of freshly produced Z-3-hexenal (Z3al). Functional groups like -NH_2_ within HALT may be responsible for the binding of Z3al. To test for this potential interaction, we tested different amino acids in various binding assays for their capacity to bind Z3al. We found a significant reduction in Z3al production in the presence of these amino acids, presumably through Schiff base formation. In a feeding assay with beet armyworm (*Spodoptera exigua*), we found a significant impact of a Z3al- or E2al-spiked artificial diet on growth and development. Together, these data indicate that the presence of Z3al in a diet can harm insect herbivores, which may help to further explain the efforts insect herbivores undertake to suppress the biosynthesis of this compound.

## Simple Summary

Green leaf volatiles (GLVs), the typical green smell of plants, are produced within seconds once the green part of a plant becomes damaged, typically by insect herbivores. These compounds induce protective defenses and can also be recognized by other plants nearby and prepare them for future herbivory. At the same time, insect herbivores have developed biochemical mechanisms to suppress GLVs, thereby reducing their potential activity. While several mechanisms have been described, we focused our efforts on the identification of potential binding sites as they may occur in the hexenal trapping molecule (HALT). Since HALT appears to bind directly to Z-3-hexenal and, thus, blocks the remainder of GLV biosynthesis, we wanted to know more about potential binding mechanisms. We found that Z3al, as an aldehyde, can form rapid bonds with amino groups, for example, in their diet. Furthermore, by exposing insect herbivores to such a modified diet, we found a significant impact on growth and development, which may explain why herbivores developed a large array of countermeasures.

## 1. Introduction

Green leaf volatiles (GLV), 6-carbon aldehydes, alcohols, and esters, are produced by most plants in large quantities upon mechanical damage, mainly caused by biotic and abiotic stresses like insect herbivores and necrotrophic pathogens, as well as cold, drought, and extreme temperatures [1,2]. These volatile compounds, when perceived by undamaged parts of the same plants or neighboring plants, have been found to induce protective responses against these threats, either by directly activating protective gene expression or by priming plants against these threats, which results in a stronger and/or faster response when these actually ensue [3,4]. Over the last 20 years, research has provided extensive evidence to support these roles of GLVs in the protection of plants and has demonstrated their effectiveness in plant-insect interactions [1,3,4,5,6].

The biosynthesis of GLV is generally well established [1]. A lipoxygenase (LOX10 in maize [6]) inserts molecular oxygen in position 13 of linolenic acid, resulting in 13-hydroperoxy linolenic acid. A hydroperoxide lyase (HPL) then cleaves off a 6-carbon unit as Z-3-hexenal (Z3al). Both enzymes are located within the chloroplasts, and these reactions happen within seconds after mechanical damage to the leaves. The aldehyde is then reduced to (*Z*)-3-hexenol and esterified to the acetate by enzymes in the neighboring undamaged leaf cells, which requires a supply of NADPH and acetyl-CoA [7].

The aldehyde can also be isomerized enzymatically and spontaneously to (*E*)-2-hexenal (E2al). Plant *Z*3:*E*2 isomerases from pepper [8] and cucumber [9] have been identified. Like the production of Z3al, the isomerization to E2al occurs within the cell debris at the wounding site [10]. E2al can also be reduced and esterified, similar to the *Z*-3 isomer. However, not all plants seem to have an isomerase, including maize, and even those with an isomerase still produce large quantities of the *Z*-3 isomer [10]. Nonetheless, the transformation of the *Z*-3 and *E*-2 aldehydes into their reduced forms is considered essential because of their potential toxicity to eukaryotic and prokaryotic cells [7,11].

While many plants can produce these compounds in large quantities upon damage [10], it has been found that insect herbivores are very effective in suppressing and/or modulating the release of these compounds when actively feeding on plants [12,13,14,15,16,17]. Several mechanisms have been identified including enzymatic deactivation of the substrate for GLV biosynthesis by fatty acid dehydratase (FHD) [15], isomerization of Z3al into E-2-HAL by an insect isomerase [12,13,16,17], and the presence of a small yet to be identified heat stable hexenal trapping molecule (HALT) [16,17]. To date, 10 caterpillars from different clades have been investigated for these suppressing mechanisms and all have been found to have at least one, but often two or all three of these mechanisms present in their saliva, thereby effectively reducing the accumulation and release of Z3al from damaged plant material by more than 90% [17]. This has raised the question of why insect herbivores possess this ability. Several hypotheses have been proposed, the main one being the general elimination of GLVs as a damage-induced molecular pattern (DAMP), which can activate defenses in systemic parts of the damaged plant but also in neighboring plants by targeting the first product of the biosynthetic pathway.

Lipid peroxidation products like Z3al and E2al are aldehydes and, as such, very reactive. They can form stable bonds with different chemical groups, including amines and thiols, leading to covalent modifications with significant consequences for the organisms. These groups can be found in proteins, but also in DNA and lipids. In the animal system, lipid-peroxidation products induced by cellular stress have long been described to significantly affect cellular signaling [18,19]. The two major peroxidation products that have been characterized to alter the physiology of animal cells are malondialdehyde (MDA) and 4-hydroxy-2-nonenal (HNE). Both were found to act as active signals that alter gene expression and cell survival but also have cytotoxic effects through the inhibition of certain gene expression and the promotion of cell death [18,19]. The specific activity of these compounds seems to depend on the concentration. Among the genes that were found to be activated by HNE, for example, are mitogen-activated protein kinases (MAPK) and other regulatory proteins. MDA forms stable Schiff bases with amino- and other nucleophilic groups, while HNE mainly forms Michael adducts with thiol and amino groups. These adducts have also been found to alter the biological activity of different regulatory proteins in animal systems. However, while these modifications are established in animal cells, it has to be noted that only very few sites, generally located on the surface of proteins, have been identified as being modified, suggesting that other yet to be identified regulatory factors are also involved in the process [20,21,22]. Regardless of the findings in animal cells, no such system has been described for plants. Only certain reactive electrophile species (RES), to which α,β-unsaturated carbonyls belong, were found to activate specific but limited gene expression, but the specific mechanism by which this is regulated still remains unknown [23,24].

**Figure 1:**
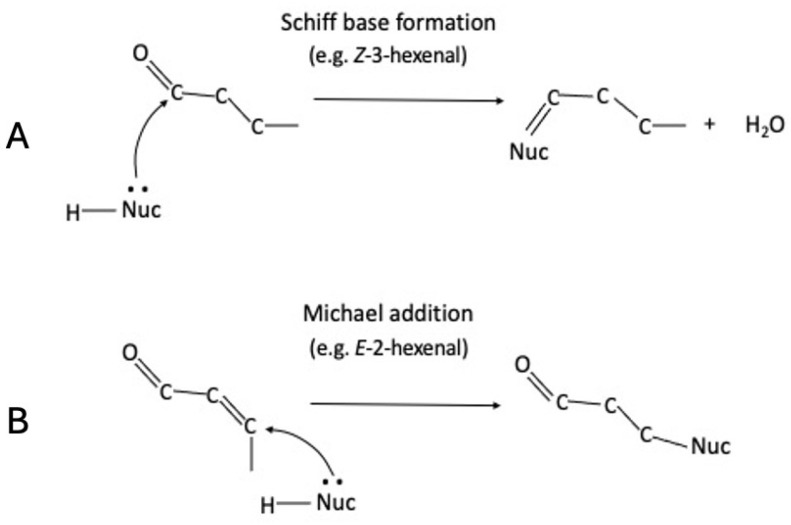
Simplified reaction schemes of different aldehydes with nucleophiles. Schiff base formation with Z-3-hexenal (**A**) and Michael addition with E-2-hexenal (**B**) with a nucleophile are likely mechanisms.

As reported above for MDA, Z3al, as the major aldehyde GLV released by damaged maize leaves, is also likely forming Schiff base conjugates with nucleophilic groups in cellular molecules, including amino groups in proteins as well as in individual amino acids, and also in nucleic acids and lipids (Figure 1A). Likewise, E2al as an α,β-unsaturated carbonyl can also bind to amino- and sulfate groups in proteins and other molecules, but by a different mechanism (Figure 1B). Here, a Michael addition is more likely, resulting in a very different kind of hexenylation when compared to what has been postulated for Z3al. The oxo group, for example, remains unchanged during the Michael addition and would create a more polar product. However, since maize does not normally produce larger quantities of E2al, we focused our efforts on Z3al. We characterized the efficiency of the reaction as well as its potential impact on insect herbivores, which may help to explain why these have developed an impressive array of countermeasures aimed at reducing the production of Z3al during herbivory. We focused our studies on the potential formation of Z3al conjugates with various amino acids with different types of amino groups and analyzed their effects on Z3al production and conjugation. Furthermore, we investigated how the formation of hexenylated compounds would affect herbivore performance. The results demonstrate the potential of selective hexenylation as a regulatory factor in plant-insect interactions.

## 2. Materials and Methods

### 2.1. Chemicals

(*E*)-2-hexen-1-al (*E*2al) and (*Z*)-3-hexen-1-al (neat) were provided by Bedoukian (Bedoukian Research, Danbury, CT, USA). (*Z*)-3-hexen-1-al in triacetin (50%) was purchased from Sigma-Aldrich (St. Louis, MO, USA) and mainly used for amino acid interactions. Amino acids and dithiothreitol (DTT) used in this study were also purchased from Sigma Aldrich. All solvents used were analytical grade.

### 2.2. Plant Material and Insect Herbivores

Maize (*Zea mays*) and mung bean (*Vigna radiata*) plants were grown in Sungro Horticulture Professional Growing Mix (Sun Gro Horticulture Canada Ltd., Seba Beach, AB, Canada) in a growth chamber under a 12 h photoperiod at 26 °C with 60% relative humidity. Light intensity was set to ca. 150 μmol m^2^ s^−1^. Plants were used at different developmental stages. For all analyses individual leaves samples from at least 3 different plants were cut, placed in a 2 mL screw cap vial, and immediately frozen in liq. N_2_. Beet armyworm (*Spodoptera exigua*) eggs were purchased as eggs from Benzon Research (Carlisle, PA, USA) together with their artificial diet and reared under regular laboratory conditions (24 °C, 12 h day/night under artificial light). Regurgitant was collected as described in [2]. Fresh regurgitant was then diluted in KPi buffer (100 mM, pH9) and stored at −80 °C until use. *Trichoplusia ni* (T. ni) was collected on cabbage patches in the San Antonio Botanical Garden and fed on Chinese cabbage until regurgitate collection.

### 2.3. Analysis of Green Leaf Volatile Production in Damaged Leaf Tissue

To analyze damage-induced aldehyde GLV production, we homogenized the individual leaf samples containing approximately 15–30 mg of leaf tissue by following the general method described in [10] with a similar general setup also being used by [25,26]. To test for the inhibitory effects of caterpillar regurgitant on GLV production, either 5 μL of 100 mM phosphate buffer, pH 9, or 5 μL of caterpillar regurgitant prepared as described above were added to the plant material together with Zirmil homogenizing beads (app. 1 mm in diameter). Tubes were then capped and homogenized in a Precellys tissue homogenizer (MO BIO Laboratories, Carlsbad, CA, USA) at 6000 shakes per minute for 25 s. The 2 mL microcentrifuge tubes were unscrewed without removing the cap and immediately dropped into a 30 mL glass container while also releasing the cap into the glass container to minimize losses in volatiles. The glass containers were immediately capped. Volatile emissions were collected immediately from the tissue homogenate by inserting a volatile collection filter packed with 30 mg Hayesep Q absorbent (Supelco, Bellefonte, PA, USA) coupled to a vacuum at 0.3 L/min for 1 h as described previously [10,22,23,25,26]. Filters were then removed and eluted with 150 µL dichloromethane, and 1000 ng of internal standard (nonyl acetate) was added. The analysis of damage-induced GLV production was performed on a Varian 3900 gas chromatograph coupled to a Varian Saturn 2200 mass spectrometer equipped with split–splitless capillary injector systems in electron impact mode (EI). Injection volume was 1 µL. The data collection, storage, and subsequent analysis were performed by using the Varian MS Workstation software (Version 6.6). Helium at a constant flow rate of 1 mL/min was used as a carrier gas. The analyses of volatiles were performed on a fused silica capillary column (Equity™ 30 m × 0.25 mm inner diameter with a 0.25-µm-thick film of bonded methyl silicone (Sigma-Aldrich, St. Louis, MO, USA)). The GC was programmed as follows: 40 °C for 2 min, then at 15 °C/min to 250 °C. All of the injections were made in the split-mode (1:20 split ratio). Compounds were identified by comparison to authentic standards (retention time and fragmentation). Due to the strong co-elution of Z3al and nHal, we used a selected ion (*m*/*z* 56) for stronger separation of the two compounds within a single peak area [26]. We then estimated the percentage of this ion in both compounds and used the calculated multiplication factor to determine the precise peak area for Z3al.

To test for the inhibitory effects of different amino acids and imidazole on GLV production, we followed an identical protocol. Amino acids were selected based on the presence of different nitrogen groups and other varied characteristics, with the expectation of finding different reactivities in our assays. Proline contains a secondary amine with a pKa of 10.6. Arginine contains 4 nitrogen atoms in different configurations and is very basic (pH 11.4) in an aqueous solution. Glutamine contains a second amino group in the side chain, while leucine and glycine only have one. Imidazole was initially used as a positive control due to its 5-membered aromatic heterocycle containing two nitrogen atoms, which appeared to be very reactive in the first assays we performed. Individual amino acids and imidazole were added to the assay in 5 μL aliquots of a 1 M solution in water. Aside from imidazole, we used proline, arginine, glutamine, leucine, and glycine for our assays. To test for the reactivity of -SH groups, we added dithiothreitol (DTT) similarly. GLV production was then assayed as described above by GC/MS. The efficiency of the reaction was then calculated as the amount of Z3al that was reduced in the presence of imidazole, individual amino acids, and DTT.

### 2.4. Analyzing the Reaction of Individual Amino Acids with Z-3-Hexenal

To further analyze the interaction of Z3al, we performed a spectrophotometric assay to monitor the reaction. Our assay contained 400 μL of H2O, 200 μL of KPi buffer, 200 μL of a 100 mM amino acid solution, and 1 μL of pure Z3HAL. The samples were mixed in a 1 mL cuvette quickly and covered with a cap to avoid evaporation of Z3al. We analyzed the reaction at pH 6 and pH 9 on a Varian Cary WinUV 50 spectrophotometer (Palo Alto, CA, USA). Samples were mixed quickly and thoroughly, and the reaction was monitored at 265 nm for a period of 3 min. The formation of a C=N double bond during the Schiff base reaction is the likely reason we were able to follow the reaction at the listed wavelength.

### 2.5. Effects of Hexenylated Diet on Growth and Development of Beet Armyworm

Commercially available insect diet (Benzon Research, Carlisle, PA, USA) was used for these experiments. The diet was first prepared as recommended by the supplier by adding it to boiling deionized water. Since it is agar-based, we allowed it to cool down to approximately 40 °C. We then added either pure Z-3-hexenal or E-2-hexenal to the diet at a final concentration of 1 mg/mL and mixed it thoroughly. To compensate for losses due to the relatively high temperature (≥40 °C) at the time of mixing in the aldehydes, as well as being stored for 24 h, Z3al and E2al were added at these higher concentrations. Later analyses revealed that the actual amount of both compounds was significantly lower and at levels usually found in low GLV producers [10]. The diet was then covered with clingwrap and immediately placed in a refrigerator. The control diet was prepared as recommended by the supplier without any further additions. All feeding experiments started 24 h after the preparation of the diet. Every other day, the diet was replaced, and weight gain, frass, and developmental stages of the caterpillars (caterpillar, onset of pupation, pupa, and adult) were monitored. With the onset of pupation, development was monitored every day. At least 20 caterpillars (1st instar) were used per experiment. All experiments were repeated at least 3 times.

### 2.6. Statistical Analysis

At least 3 biological replicates were performed per experiment. Averages and standard deviation (SD) were calculated for all samples. For pairwise comparisons, Student’s *t*-test was used (Microsoft Excel, Version 16.88), while for multiple comparisons, one-way ANOVA and Tukey’s test were applied (JMP statistical software, Version 17.2).

## 3. Results and Discussion

### 3.1. Amino Acids and GLV Production

**Figure 2:**
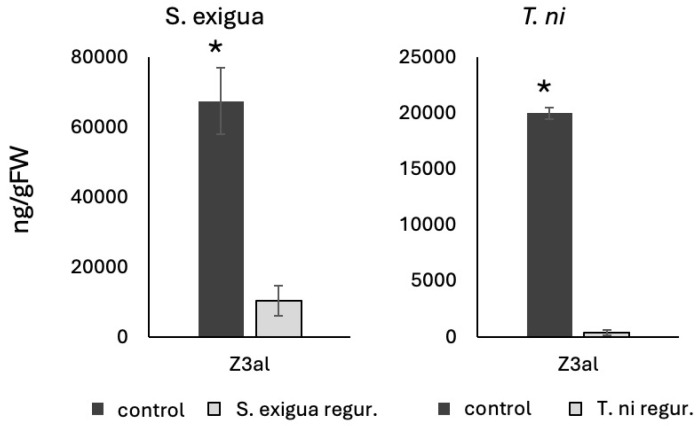
Inhibition of Z-3-hexenal production by caterpillar regurgitant. Z3al production in the presence of regurgitant (regur.) from *Spodoptera exigua* (*S. exigua*) and *Trichoplusia ni* (*T. ni*) is significantly reduced when compared to control samples. Error bars represent standard deviation. * denotes significant differences (*t*-test, *p* ≤ 0.05) between samples.

We first analyzed the effects of fresh caterpillar regurgitant on the GLV production in maize leaf tissue. Adding 5 μL of regurgitant from BAW reduced the amount of Z3al produced in the damaged tissue by 85% (from 67,485 ± 9499 ng/g fresh weight in control samples to 10,437 ± 4303 ng/g fresh weight) (Figure 2). Likewise, fresh regurgitant from *T. ni* reduced the amount of GLV produced from fresh leaf tissue from 20,010 ± 544 ng/g fresh weight to 411 ± 210 ng/g fresh weight, corresponding to a more than 90% reduction (Figure 2). It has to be stated that in the experiment with *T. ni* the overall capacity of the plant tissue to produce Z3al used for this experiment was much less (~20,000 ng/gFW) than what was used for the BAW regurgitant analysis (~70,000 ng/gFW) (Figure 2), which may have allowed for a more significant reduction in Z3al in the *T. ni* assay. Also, in contrast to previous studies [16,17], where a neutral pH was used, we opted for a more natural pH at around 5–6 for our analysis of damaged plant tissue. This was in accordance with previous results in our lab [25], where an optimum production capacity for GLV was found to be in this range and corresponds to what is found in damaged maize leaf tissue. Despite these differences, the results again clearly demonstrated the efficiency of the caterpillar regurgitant to inhibit the production of GLV in damaged leaf tissue from maize. While at least two mechanisms have been found to be abundant in either BAW and *T. ni* regurgitant, including FAD and HALT [16,17], our main focus has still been on HALT as a molecule that can bind to Z3al directly, thereby reducing the ability to produce larger quantities of others.

GLVs, including Z-3-hexenol, which has been proposed to be the main bio-active compound within this group of molecules [27]. Since amino acids have a variety of amino groups, which can react with aldehydes like Z3al, we tested six amino acids in a first approach to characterize potential HALT-reactive groups that may interfere with Z3al production.

### 3.2. Inhibition of Z3al Production by Specific Amino Acids

**Figure 3:**
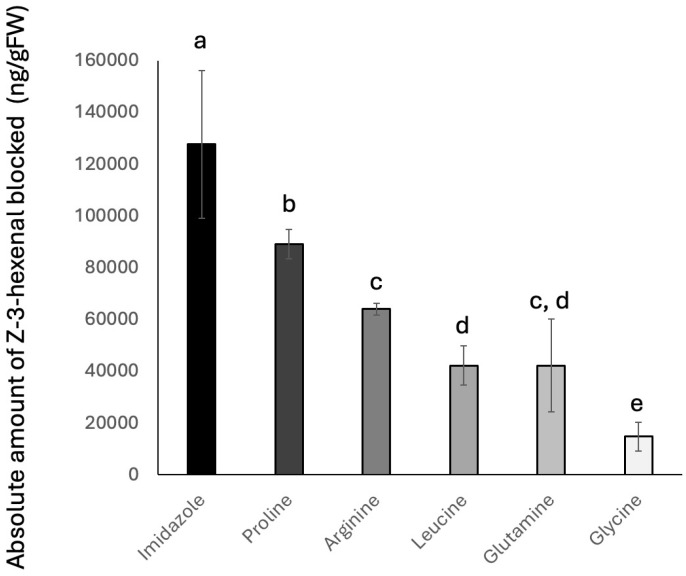
Inhibition of Z-3-hexenal (Z3al) production in maize (*Zea mays*) leaf tissue by amino acids. All amino acids were used at the same concentration. Shown are the amounts of Z-3-hexenal that are blocked in the presence of the amino acids and imidazole. Different letters above each bar indicate statistical differences determined by ANOVA analysis, followed by Tukey tests where appropriate (*p* < 0.05). *N *≥ 3, error bars represent standard deviation.

We selected a variety of amino acids and tested their ability to block Z3al biosynthesis in our capacity assay. In particular, we used arginine, glutamine, glycine, leucine, and proline in a similar assay as described above for the caterpillar regurgitant. We further added imidazole due to its two amides in a five-ring system. All amino acids and imidazole were used at the same concentration, and plant material was also used from plants with similar capacities to produce Z3al. Results are shown in Figure 3. We found that imidazole had the highest activity in reducing Z3al production by quenching 127,644 ± 28,453 ng Z3al per g fresh weight. Among the amino acids, proline was most efficient, quenching 89,109 ± 5576 ng/g fresh weight. Arginine quenched 63,999 ± 2352 ng Z3al per gram fresh weight, while Leucine and glutamine were less active (42,228 ± 7570 ng/fresh weight and 42,179 ± 17,895 ng/g fresh weight, respectively). The least active amino acid in our assay was glycine, with only quenching 14,755 ± 5649 ng/g fresh weight. We assume that the pKa of the amino group(s) is the essential factor in the reactivity of these amino acids with Z3al. Proline, for example, has a much higher pKa value (10.6) for its secondary amide than leucine, glutamine, and glycine (9.6, 9.13, and 9.6, respectively) for their primary amide. However, arginine does not seem to follow this trend. Based on the fact that it contains four nitrogen atoms in different configurations, with two of those being mainly active, it is still unclear to us how this amino acid fits into this scheme. However, arginine is also a strong base and may, as such, interact with the reaction by increasing the pH, which in itself reduces the activity of the Z3al-producing system [25].

**Figure 4:**
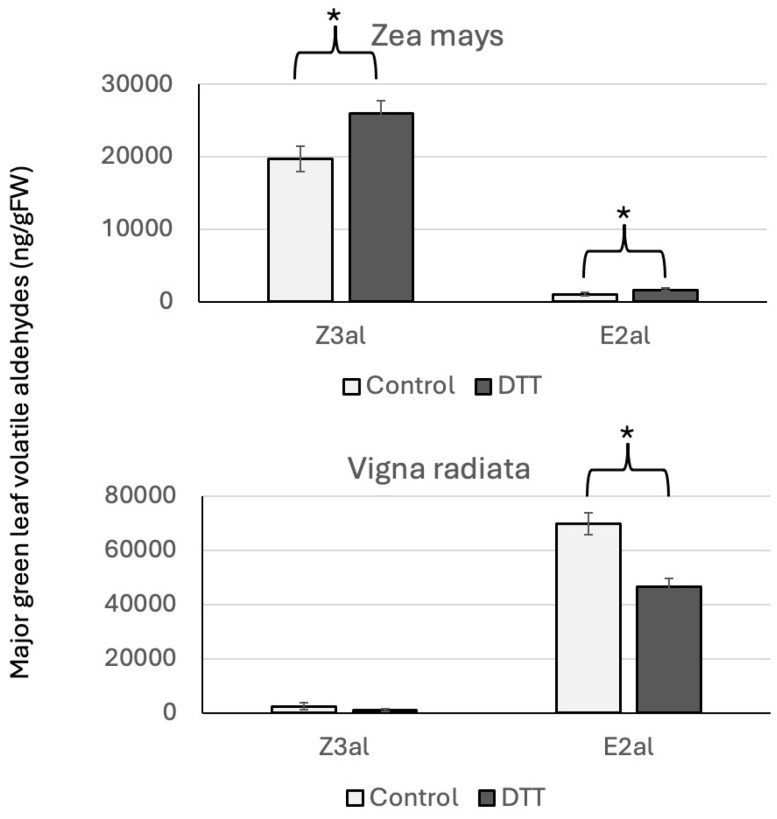
Effects of dithiothreitol on aldehyde GLV production in maize (*Zea mays*) and mung bean (*Vigna radiata*). * denotes significant differences (*t*-test, *p* ≤ 0.05). *N *≥ 3, error bars represent standard deviation. DTT, dithiothreitol; Z3al, Z-3-hexenal; E2al, E-2-hexenal. Note that maize is a Z3al emitter while Vigna predominantly produces E2al.

We further investigated the effects of dithiothreitol (DTT) as a thiol-containing molecule on Z3al and E2al production in crushed plant material from maize and mung bean (*Vigna radiata*) leaf segments. While maize is predominantly a Z3al producer, mung bean mainly produces E2al during its early developmental stages. Using DTT would therefore allow us to test the differential effect of this compound on the production of both GLV aldehydes as well as the potential type of reaction. As mentioned above, Z3al is expected to form Schiff bases with amino groups, while E2al should predominantly form Michael adducts with thiol groups due to it being an α,β-unsaturated carbonyl [18,19,23,24]. Accordingly, we found that in the mung bean assay, the addition of DTT did decrease the amount of E2al (from 69,813 ± 2351 ng/g fresh weight in the control assays to 46,588 ng/g fresh weight in the DTT assay) (Figure 4). For maize, we found that adding DTT did increase the production of Z3al. Control samples produced 19,684 ± 1792 ng/g fresh weight. However, with the addition of DTT, this was brought up to 25,972 ng/g fresh weight (Figure 4). While this seems to confirm that α,β-unsaturated carbonyls prefer to react with thiol groups, it is still unclear what caused the increase in Z3al production from maize tissue. Changes in the redox potential as well as interference with disulfide bridges may be responsible for this. It is, however, clear that Z3al production is not negatively affected by the presence of thiols, and thus, it is rather unlikely that such a group is abundant in HALT since it directly interferes with Z3al [17].

### 3.3. In Vitro Assays for Schiff Base Formation

**Figure 5:**
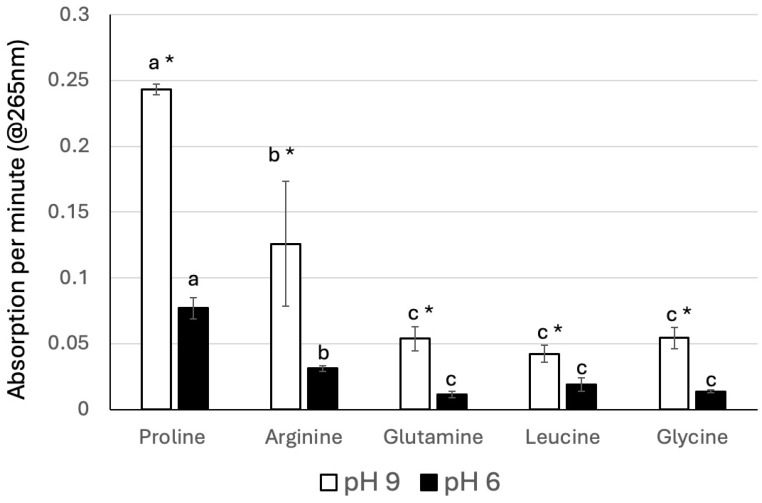
Reaction of Z-3-hexenal with selected amino acids. All amino acids were used at the same concentration as described in the Materials and Methods. *N *= 3, error bars represent standard deviation. Different letters above each bar indicate statistical differences determined by ANOVA analysis, followed by Tukey tests where appropriate (*p* < 0.05). * Denotes significant differences between pH 9 and pH 6 assays (*t*-test, *p* ≤ 0.05).

To further test for the differential reactivity of amino acids with Z3al, we developed an assay that monitors changes in the absorption of the two reactants, the amino acid and Z3al. We found that during the reaction, a change in the absorption at 265 nm occurred, presumably due to the formation of a C=N double bond during the Schiff base reaction. We also tested the reactivity at two different pH levels based on the natural conditions in which these reactions can occur. Insect regurgitant is usually very basic with a pH around 9, while damaged leaf material is between pH 5 and 6. We therefore used both pH for our assays, where we also focused on the reactivity of actual amino acids. Proline was again the most effective amino acid with an average change in absorption of 0.243 ± 0.0042 per minute at pH 9 (Figure 5). At a pH of 6, the reaction was significantly slower (0.077 ± 0.0081 per minute). Compared to proline, arginine was less reactive despite having more amino groups, with an average absorption of 0.126 ± 0.047 per minute at pH 9 and 0.031 ± 0.0021 at pH 6. Glutamine, leucine, and glycine were very similar in their reaction speed (0.0539 ± 0.0092, 0.0423 ± 0.0064, and 0.0543 ± 0.0081, respectively, at pH 9, and 0.0113 ± 0.0026, 0.0188 ± 0.005, and 0.014 ± 0.00096 at pH 6, respectively) (Figure 5). Overall, these data confirm our finding regarding the inhibition of Z3al production in damaged leaf tissue by these amino acids. The results further provide important insights into the potential regulation of the processes involving HALT. In the caterpillar regurgitant, the more basic pH would promote the reaction of amino groups with Z3al, in general, and may, as such, already contribute to the effective binding of these two compounds. However, in the plant material, the reaction would be significantly reduced due to the much more acidic pH, which may help the plant to (a) continue to produce Z3al, and (b) reduce reactions of Z3al with its own amino groups, thereby avoiding potential negative interactions and consequences. This is therefore another example of how pH may impact the interaction of plants and their insect herbivores. Z3al biosynthesis as such strongly depends on the pH and has a relatively narrow window, in which the damaged tissue can produce the compound efficiently [25]. At the same time, insect regurgitant with its much more basic pH [17] already blocks the biosynthesis and also allows for a much more efficient binding of Z3al to HALT. One can therefore assume that the preferential use of pH may be another co-evolutionary factor that regulates the interaction of plants and their insect herbivores.

### 3.4. Effects of Z-3- and E-2-Hexenylated Diet on BAW Growth and Development

**Figure 6:**
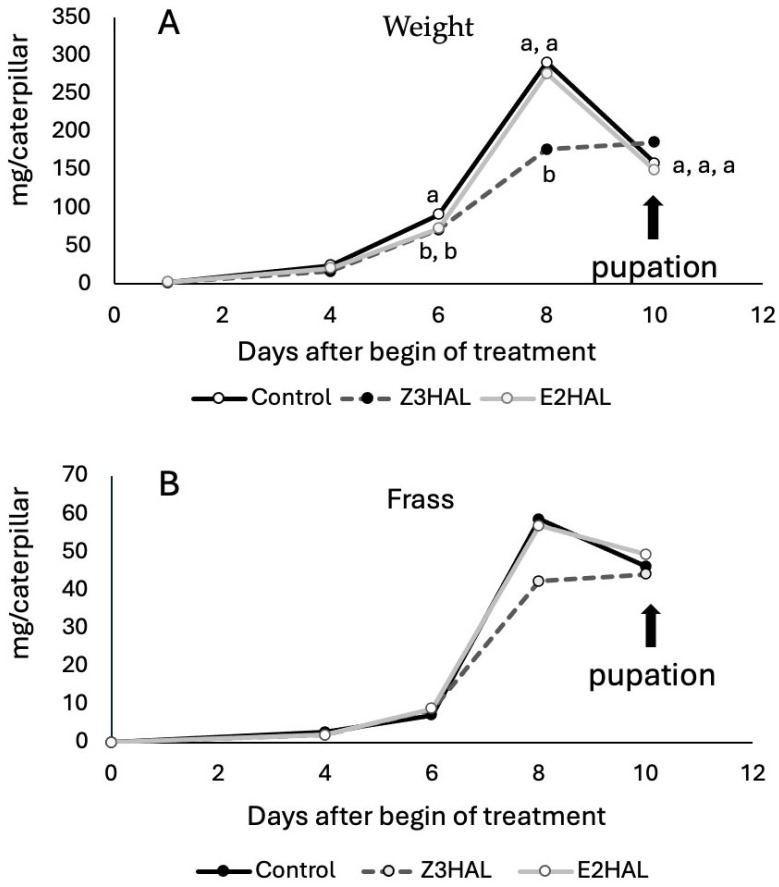
Effect of Z3al and E2al-spiked diet on the growth of beet armyworm (BAW, Spodoptera exigua). (**A**) Weight gain of beet armyworm (BAW, *Spodoptera exigua*) (neonates at day 0) on artificial diet spiked with Z-3-hexenal (Z3al), E-2-hexenal (E2al), and control diet over 10 days. Different letters above each bar indicate statistical differences determined by ANOVA analysis, followed by Tukey tests where appropriate (*p* < 0.05). (**B**) Frass produced by caterpillars over the treatment period. Note that total frass was collected and then divided by the number of caterpillars. Therefore, no statistics could be applied.

Since Z3al can directly react with an array of amino groups, we wanted to study the effects of Z3al on insect herbivore performance by adding it to the artificial diet. Studies on plant mutant lines, which are depleted in this pathway, have shown that strongly reduced levels of GLV are beneficial for insect performance on these plants when compared to normal GLV-producing wild types. However, insect herbivores also apply elicitors abundant in their saliva to the plant, which in turn, activate a wide array of defensive measures [28]. These may then interfere with the assessment of herbivore performance on both GLV-depleted and normal plants. We therefore decided to perform an assay that is free of the activation of other plant defensive pathways. Since BAW caterpillars are normally reared on an artificial diet, we added Z3al and E2al to the diet. The control diet was without the addition of any other compound. After letting the diet rest for 1 day, we then provided it to first instar BAW caterpillars. The weight and developmental status of the caterpillars were monitored every second day. Also, the frass of the caterpillars was measured. Over the first 4 days, no significant differences were found (Figure 6A). Weight was statistically similar in all three groups of BAW caterpillars. However, beginning on day 6, we found significant differences in the weight of the caterpillars. Control caterpillars showed more weight at this time point (91.1 mg/caterpillar), while both caterpillars on the Z3al- and E2al-containing diets were lower in weight (70.9 and 72.6 g/caterpillar, respectively). On day 8, caterpillars on control and E2al-diet were similar with regard to their weight gain (301.9 and 275.8 mg/caterpillar, respectively). At the same time, caterpillars on the Z3al diet were significantly lower in weight (176.7 mg/caterpillar). On day 10, which marked the beginning of pupation, all three treatment groups had the same weight again (Figure 6A). However, while the control and E2al group went through a significant weight loss between days 8 and 10, just before the onset of pupation, the Z3al-treated caterpillars did not go through this process but rather entered pupation with their maximum weight. Pupae in all groups did not show any differences in weight when analyzed on day 15

The exact same trend was found in the amount of frass that accumulated over the 2-day feeding periods (Figure 6B). Since we measured only total frass divided by the number of caterpillars within each treatment group, we did not perform a statistical analysis. However, the similarities between weight gain and frass production indicated that Z3al-fed caterpillars either consumed far less diet material compared to the other two groups or suffered from less effective digestion. Overall, the results showed that Z3al-diet had a significant negative effect on caterpillar weight, while E2al only seemed to affect very young caterpillars up to day 4; however, from day 6 on, their weight gain was similar to that of control caterpillars.

**Figure 7:**
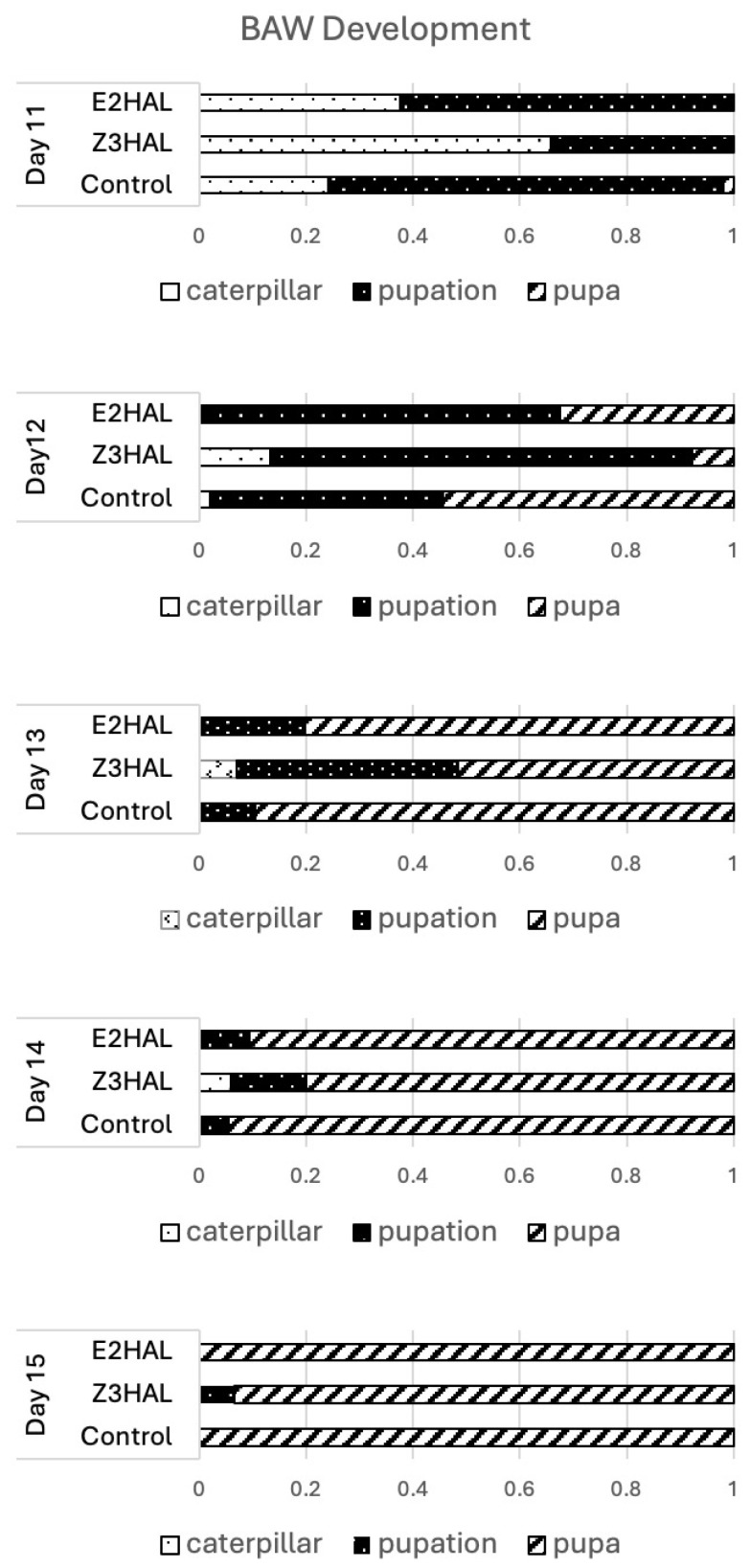
Effects of artificial diet spiked with Z-3-hexenal (Z3al) and E-2-hexenal (E2al) on the development of BAW caterpillars. Twenty caterpillars were used for each assay in each individual experiment. The combined results of 3 individual experiments are shown. The x-axis shows the percentages of the different stages. The statistical analysis is shown in Table S1 (ANOVA with Tukey HSD).

From day 10 after the start of the experiment, we focused mainly on the insect development in the three treatment groups (Figure 7). Between day 11 and day 12, we found no significant differences between the control group and the E2al group regarding the onset of pupation. However, on day 12, a shift was observed between these two treatments. While the number of BAW at the caterpillar stage remained similar between control and E2al-treated, the onset of pupation changed. E2al-treated caterpillars developed from here on slower and more like the Z3al-treated ones. In contrast, caterpillars reared on the Z3al diet were significantly delayed in their development right at the beginning of this developmental process (Table S1 for statistical details). While 74% of caterpillars in the control group were already in the process of pupation, only 34% of the Z3al group had entered this stage. On day 13, 90% of control caterpillars were already pupated, but only 51% of the Z3al group. On day 15, all of the control group caterpillars as well as the E2al caterpillars were pupated; however, 7% of the Z3al group were still in the process of pupation (Figure 7). It appears as if a diet spiked with E2al does not deter caterpillars from consuming similar to those of the control group, but it still has a negative effect on later developmental processes. This may in part explain why certain caterpillars possess an isomerase in their saliva [12,13,16,17]. And while this isomerase is not specifically aimed at the conversion of Z3al to E2al [29], it may still help to avoid the more immediate deterring effects of Z3al. However, it does not seem to reduce the negative effects of aldehyde GLV on the development of BAW caterpillars during metamorphosis, as shown for E2al herein.

**Figure 8:**
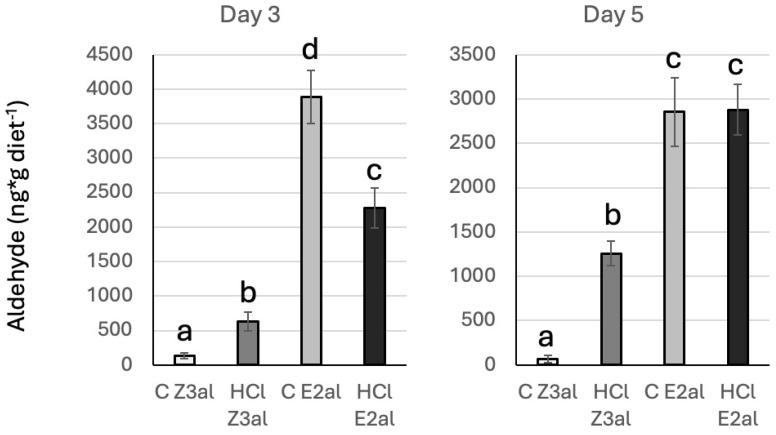
Analysis of Z-3-hexenal (Z3al) and E-2-hexenal (E2al) in the artificial diet. Samples were analyzed on day 3 and day 5 after the respective aldehyde was added to the diet. Different letters above each bar indicate statistical differences determined by ANOVA analysis, followed by Tukey tests where appropriate (*p* < 0.05). C, control, HCl, hydrolyzed with hydrochloric acid.

As the amounts of Z3al and E2al added to the respective diet were higher than what is usually found in nature (1 mg/mL diet compared to a maximum of 100 ug/g fresh weight in older maize leaves [26]), we monitored the actual amounts of Z3al and E2al present in the diet by GC/MS 3 and 5 days after these compounds were mixed in. Surprisingly, we only found 128.2 ng/g diet at day 3 and 63.9 ng/g diet at day 5 for the Z3al-spike diet (Figure 8). For E2al, we found 3883.2 ng/g diet on day 3 and 2854.3 ng/g diet on day 5. We then hydrolyzed the same samples by adding HCl at 95 °C for 1 h. After the second collection, we found 632.6 ng of Z3al per g diet and 1257.8 ng/diet on day 3 and day 5, respectively, indicating that most Z3al was conjugated to other molecules in the diet. This further indicates that Z3al, even when it is conjugated to other molecules in the diet, still has a significant effect on the growth and development of BAW caterpillars. For E2al, we found 2275.2 ng and 2880 ng per g diet at day 3 and day 5, respectively (Figure 8). While here much of the E2al was also conjugated to the diet, the effect of E2al was found to be less deterring during the active feeding phase of the caterpillars, as shown in Figure 6, and allowed those BAW caterpillars to grow similar to the control group during this phase.

The results from the feeding assays clearly show a significantly harmful effect of Z3al and E2al on caterpillar performance and provide at least some further insight as to why caterpillars have developed an arsenal of countermeasures to reduce the levels of Z3al in herbivore-damaged and ingested plant material. However, several questions still remain unanswered. First, what is HALT actually? The molecule has not yet been identified, and we can therefore only speculate about the nature and active groups in this molecule. Amides, rather than thiols, are likely candidates, but whether they are amino acid-derived or products of other pathways still remains unclear. To date, we only know that HALT is a heat-stable molecule [16], suggesting that it is not an enzymatic process that removes Z3al. Modern mass spectroscopic techniques should help to identify this molecule as well as others that may become hexenylated. For Z3al, the addition of *m*/*z* 80 or multiples thereof due to H_2_O loss in the Schiff base formation should be helpful in the characterization of these modified molecules.

If hexenylated products have a harmful effect on caterpillars, what is the fate of hexenylated HALT or other micro- and macromolecules modified in the same fashion? Are those just repellent, or do they also have physiological consequences? This might be relevant for the insect herbivore, but also for the plant itself. Hexenylated molecules in the plant cells could also be a source for the slow release of GLV over a longer period of time. As with all chemical reactions, the process of hexenylation is reversible and may provide a relatively long-term release mechanism for GLV, which may help to extend the anti-herbivore effects due to its prolonged activation of plant defense mechanisms. Further exploring this aspect may lead to a much more efficient application in agricultural and horticultural settings, but may also be relevant in the regulation of plant-insect interactions.

Likewise, aldehyde-dependent crosslinking of proteins has been repeatedly described to occur in various cell types [18,30,31], and it might be the case that Z3al serves such a function in the wound response since this is where it is mainly produced. But does this also have consequences for the herbivore, for example, by making the ingested food less digestible? These and probably many more questions cannot be answered at this time, but the presented study provides a starting point for future research into the role of aldehyde GLV and how they may affect proteins, lipids, nucleic acids, and other nitrogen-containing molecules in plants and their insect herbivores, the consequences of which still need to be elucidated.

## 4. Conclusions

By studying the interaction of various amino acids with Z3al, we identified novel parameters that may be involved in the modulation of plant-insect interactions. Aldehyde GLV has significant direct effects on caterpillars and plants through the hexenylation of distinct targets with yet to be identified physiological consequences. We show that certain types of amino groups seem to react efficiently with Z3al, which may result in covalent modifications of amino acids and consequently proteins with a potential to alter their functionality, but also their digestibility. But while we did find a significant effect of such modified diets on the growth and development of caterpillars, we still know very little about the actual effects of these modifications on plants. The potentially negative effects of such a hexenylated diet provide an additional reason as to why insect herbivores have developed effective countermeasures to reduce aldehyde GLV.

## Data Availability

The original contributions presented in this study are included in the article/Supplementary Material. Further inquiries can be directed to the corresponding author.

## Supplementary Materials

The following supporting information can be downloaded at https://www.mdpi.com/article/10.3390/insects16060582/s1, Table S1: Results of Statistical Analysis.
