# Supplementary material for: Reactivity of Z-3-Hexenal with Amino Groups Provides a Potential Mechanism for Its Direct Effects on Insect Herbivores
Source: Insects. 2025 May 31;16(6):582. doi: 10.3390/insects16060582 (PMC12193613; doi:10.3390/insects16060582)
Supplement: Supplementary file 1 [file insects-16-00582-s001.zip › insects-3638955-supplementary.pdf]

Table S1: Results of Statistical Analysis

Anova with Tukey HSD														
**Day 11**			**Day 12**			**Day 13**			**Day 14**			**Day 15**		
**Caterpillars**			**Caterpillars**			**Caterpillars**			**Caterpillars**			**Caterpillars**		
treatments	Q statistic	p-value	treatments	Q statistic	p-value	treatments	Q statistic	p-value	treatments	Q statistic	p-value	treatments	Q statistic	p-value
Control vs Z3al	4.6239	**0.038918**	Control vs Z3al	7.6862	**0.003857**	Control vs Z3al	3.3817	**0.116892**	Control vs Z3al	3	0.165075	Control vs Z3al	0	0.899995
Control vs E2al	0.2531	0.899995	Control vs E2al	2.1617	0.343776	Control vs E2al	0	0.899995	Control vs E2al	0	0.899995	Control vs E2al	0	0.899995
Z3al vs E2al	4.877	**0.031406**	Z3al vs E2al	9.8479	**0.001059**	Z3al vs E2al	3.3817	**0.116892**	Z3al vs E2al	3	0.165075	Z3al vs E2al	0	0.899995
**Pupation**			**Pupation**			**Pupation**			**Pupation**			**Pupation**		
treatments	Q statistic	p-value	treatments	Q statistic	p-value	treatments	Q statistic	p-value	treatments	Q statistic	p-value	treatments	Q statistic	p-value
Control vs Z3al	5.3051	**0.022071**	Control vs Z3al	15.4081	**0.001005**	Control vs Z3al	5.3067	**0.02204**	Control vs Z3al	8.3603	**0.002511**	Control vs Z3al	3.005	0.164336
Control vs E2al	0.8139	0.82505	Control vs E2al	13.6751	**0.001005**	Control vs E2al	5.134	**0.025372**	Control vs E2al	10.9327	**0.001005**	Control vs E2al	0	0.899995
Z3al vs E2al	6.119	**0.01167**	Z3al vs E2al	1.733	0.483274	Z3al vs E2al	0.1728	0.899995	Z3al vs E2al	2.5724	0.24162	Z3al vs E2al	3.005	0.164336
**Pupa**			**Pupa**			**Pupa**			**Pupa**			**Pupa**		
treatments	Q statistic	p-value	treatments	Q statistic	p-value	treatments	Q statistic	p-value	treatments	Q statistic	p-value	treatments	Q statistic	p-value
Control vs Z3al	1.7321	0.48361	Control vs Z3al	14.7975	**0.001005**	Control vs Z3al	8.9658	**0.001742**	Control vs Z3al	11.1093	**0.001005**	Control vs Z3al	3.0487	0.157968
Control vs E2al	1.7321	0.48361	Control vs E2al	9.8907	**0.001033**	Control vs E2al	6.4585	**0.009058**	Control vs E2al	8.6442	**0.002112**	Control vs E2al	0	0.899995
Z3al vs E2al	0	0.899995	Z3al vs E2al	4.9068	0.03063	Z3al vs E2al	2.5073	0.255812	Z3al vs E2al	2.4651	0.265412	Z3al vs E2al	3.0487	0.157968

Bold numbers indicate significant differences. Z3al, Z-3-hexenal; E2al, E-2-hexenal
